# On the Suppression of Hæmorrhage in Cancer Uteri

**Published:** 1854-10

**Authors:** Thomas Thomson


*On the Suppression of Hæmorrhage in Cancer Uteri.* By Thomas Thomson, M.D.,—I have lately been attending a case of scirrhous ulceration of the os uteri, accompanied by occasional hæmorrhages of an alarming extent, to arrest which I applied pledgets of lint to the bleeding surface, and used styptics of various kinds; but the result was by no means satisfactory. About three months ago it occurred to me to employ injections of creosote; and, at the same time, to paint the ulcerated neck of the uterus with a strong emulsion of creosote and alum. This had the effect of completely controlling the hæmorrhage, as well as of destroying in the discharges all fetor, from which latter annoyance both my patient and her friends had experienced great inconvenience.

I began the treatment with a very weak emulsion, and gradually increased its strength. At first I used half an ounce of creosote to eight ounces of mucilage of gum tragacanth. A tablespoonful of this mixture was added to pint of water, and injected into the vagina, after having first well washed the passage by injections of cold water. When I could not otherwise control the hæmorrhage, I several times introduced a bivalved speculum, and painted the ulcerated surface with a small sponge saturated with a very strong emulsion, and then passed a round pledget of lint soaked in an emulsion not so strong, and pressed in on the parts. The result exceeded my most sanguine expectations.

Two gentlemen, who attended the case with me, agreed in the good results of this plan of treatment, and expressed their determination to give it a fair trial on the first opportunity.—*Med. Times.*

